# A pragmatic plan to develop community health workers as researchers and strengthen Black clinical trial enrollment

**DOI:** 10.1017/cts.2025.10221

**Published:** 2025-12-17

**Authors:** Robin Gotler, Delores Collins, Michael Matthews, Samia Marchmon, Janterria Matthews, Kurt Stange

**Affiliations:** 1 Center for Community Health Integration, Case Western Reserve University, Cleveland, OH, USA; 2 A Vision of Change Inc., Cleveland, OH, USA; 3 P5 Ventures Inc., Marlton, NJ, USA; 4 Cleveland State University, Cleveland, OH, USA; 5 Cleveland Clinic, Cleveland, OH, USA

**Keywords:** Community health workers, black people, clinical trials as topic, research subject recruitment, research personnel

## Abstract

Low enrollment of racial/ethnic minorities in clinical trials is a persistent problem. This study explores community health workers’ (CHWs) potential to increase research participation by Black people. We interviewed 12 CHWs and 12 Black community members, and after multidisciplinary analysis, held a CHW focus group to refine themes and make recommendations. Most participants mistrusted research, but many valued its potential for generativity. CHW involvement in research was seen as an opportunity to bring community relationships and context to all aspects of research, including recruitment. Participants proposed steps to build trustworthy research experiences and develop CHWs as full research team members.

## Introduction

Participation in clinical trials is a crucial step in generating new knowledge to improve population health. Despite numerous recruitment strategies and interventions, however, racial minority populations in general, and people who are Black in particular, have persistently low levels of clinical trial enrollment [1], which risks reinforcing inequities in healthcare and health [2].

At the same time, community health workers (CHWs) are increasingly involved in a wide range of health and healthcare activities [3–5]. While their involvement includes participation in research, especially community-based participatory research (CBPR) in which they hold a variety of roles, they are less likely to participate in non-CBPR research, with particularly low involvement in study planning, analysis, and dissemination [6,7]. CHWs’ limited involvement in healthcare research may constrain researchers’ insights into community issues and reduce community members’ sense of connection to the research team, a factor believed to facilitate Black clinical trial enrollment [8,9].

To shed light on barriers to Black clinical trial enrollment and the broader roles that CHWs can play in research, we interviewed Black community members and CHWs to learn, (1) how Black enrollment in clinical trials can be increased and how clinical research can be made more relevant and accessible to Black community members and, (2) how CHWs can advance both Black clinical trial enrollment and the base of healthcare knowledge by becoming established community health researchers.

## Materials and methods

In April 2024, we emailed all 65 active CHWs affiliated with A Vision of Change (AVOC), a nonprofit organization dedicated to addressing social and health issues in a predominantly Black Cleveland, Ohio neighborhood. AVOC trains and employs local community members as CHWs and is widely considered a trusted community resource. The email invited them to participate in a study designed to make clinical trial research more inclusive. Twelve CHWs responded and agreed to take part in 30–45 minute interviews with a research team member (RG). Interviews were conducted in the approximate order in which CHWs responded. At the end of the interview, CHWs were asked to recommend Black community members who might be interested in being interviewed. A research team member (RG) emailed 23 community members suggested by CHWs and by AVOC. Fourteen community members responded and agreed to participate. A total of 12 interviews were conducted, as two community members did not attend.

Two research team members (DC, RG) conducted interviews with community members. Both CHW and community member interviews explored participants’ perceptions of and feelings about medical research, clinical trials, and CHWs’ roles in research recruitment (see Appendix A). Questions were open-ended and additional detail was encouraged. CHW interviews took place on Zoom. Community member interviews took place on Zoom, in person, or by telephone depending on the interviewee’s preference. Zoom calls and in-person interviews were video-recorded and transcribed. For phone interviews, the interviewer took extensive notes capturing salient quotations verbatim. Verbal informed consent was obtained prior to each interview.

We analyzed interviews on an ongoing basis, identifying major themes until we reached saturation, the point where new information and new themes no longer emerged [10–12]. We reached saturation at 9 CHW interviews and 10 community member interviews. In each case, we conducted additional interviews with interested participants (3 CHWs and 2 community members) to verify thematic findings. We held a series of analysis meetings for CHW interviews, and later for community member interviews. Research team members read de-identified transcripts to discover themes and compared them, examining data for confirming and disconfirming evidence (immersion/crystallization process) [13]. To challenge and refine themes, we identified information-rich text and quotations (editing process) [14]. Based on commonality among identified themes, we combined the CHW and community member interview findings. Two auditors – a community health educator/nurse (JM) and a primary care researcher/family and public health physician (KS) – reviewed and validated the findings based on their experience and expertise.

CHW participants were invited to a focus group meeting. Seven attended in person and one attended on Zoom. To encourage participation and reduce single-moderator bias, we used a respondent-moderator format including two research team member moderators (DC, RG), while one CHW summarized the discussion on easel notes. The meeting provided member checking, a qualitative validation technique, with participants verifying that themes identified in the analyses matched their perceptions. In addition, they considered the roles CHWs can play in clinical research and made recommendations for policy, practice, and research.

The study was reviewed and approved by the Case Western Reserve University Institutional Review Board.

## Results

### Participant characteristics

Twelve CHWs and 12 community members participated in the study. Participants were primarily female, including one male CHW and three male community members. Twenty-three participants were Black; one CHW identified as Hispanic.

### Thematic similarities

Analyses of CHW and community member interviews yielded similar themes. Below we indicate differences where they were apparent.

### Perceptions of research and motivations to participate

Overall, CHWs were more familiar than community members with the concept of research. Both groups expressed deep mistrust of clinical research and the healthcare system (see Table 1), and many mentioned historical mistreatment and/or ongoing bias/injustice towards Black people in healthcare and more generally. Participants, particularly CHWs, felt that research should benefit not only those conducting the study but also the community in which the study takes place. In spite of their concerns, many participants recognized the value of research. Compared to CHWs, community members more often described the opportunity to serve the greater good as motivation for research participation. For both groups, financial incentives and the chance to improve their own health were additional motivators. Both community members and CHWs offered specific suggestions for overcoming mistrust and creating a positive research experience. (See Table 2.)

**Table 1.** Black community members’ and CHWs’ perceptions of research

Theme	Perception	Supporting Quotes
Mixed Feelings about Research	Research applicable to Black people is important but tempered by mistrust	There is a positive connotation to [research], but, for me, as a Black woman, it’s very scary. It’s not to be trusted… you’re gonna be playing with my life and it’s not gonna matter if I live or die (CHW 05) Black people are leery to anybody that comes… researching, helping, or doing anything that has to do with us, because the outcome usually isn’t in our best interest (Cmty 23)
	Research should be a two-way street, giving back to the community in which it takes place	Are you gonna bring [the research findings] to the community, to actually do something in the community you’re asking [to participate]? (CHW 03)
	Creating a better future, improving one’s health, and payment are key incentives for participating in research	As long as you’re true [with] me, I will be willing to do a clinical [trial]…it’s all about mankind and helping people (Cmty 13) I have asthma, so if there was a new drug for that I’d [take part in research]. I’d do something that would help me now (Cmty 17) What draws people is the money (Cmty 16)
	Clinical trial participation, especially side effects, can be difficult	‘Is this an actual medication I’m taking or a sugar pill, a real or a fake? Does it work? Will it work? What’s my side effects?’ Having those things in mind does mess with you (CHW 07)

**Table 2.** Recommendations for researchers

Theme	Recommendation	Supporting Quotes
Overcoming Mistrust	Include people who are Black on the study team	When you see someone…from a hospital with different lettering behind their name, or who may not look like me…you’re coming from a different perspective, and I don’t know how true you are with wanting to help me (CHW 12)
	Include CHWs in recruitment	We’re the link in the community… [people] come to us and say, ‘What do you think about this?’ (CHW 03)
	Create an honest, respectful, and welcoming environment	Don’t start by offering money. First tell me why the research is important, otherwise people get suspicious (Cmty 18) Be honest about why you need more people who are Black [in your study]…I think Black people want more transparency (Cmty 24)
	Don’t just talk. Listen and interact.	Come into the community and…observe and see what needs to be done (Cmty 22) Sit down with [community members]…let them tell you…their barriers… Maybe they need more than a gift card. (CHW 02)
Creating a positive research experience	Share information before, during, and after the study	[keep participants] involved in the whole process, because they feel like it’s their information (CHW 06) Give me all my results based on me participating…What did you see? And then, what do you see…based on what it is that you’re looking for? (Cmty 23)
	Be open to participants’ input	[hear] from the participants what their concerns would be at each interval… maybe they’ll give you an idea…or at least they know…“my voice is being heard” (CHW 12)
	Make sure instructions are clear and logistics are easy to manage	With this [study] I’m in now, the bus passes they were giving out expired last year… I have to call my social worker every time I have an appointment (CHW 09)
	A good experience for participants can have a ripple effect	If [community members] see a couple of people [doing something], they’ll think about it, but if they see more and more people, then they’ll come. It’s like follow the leader in my community. (CHW 01)

### CHWs as research team members: Pragmatic steps

There was consensus among community members that CHWs, based on their trusted relationships and community knowledge, can play key roles in recruiting people who are Black for clinical research. CHWs agreed and went further, proposing a role as full participants in planning, implementing, and analyzing clinical research. As partners in all stages of research, CHWs envisioned themselves bridging context, communication, and other potential barriers, thus helping researchers and community members better understand and meet one another’s needs. Their vision includes bringing on-the-ground perspectives to study planning/design, data collection, and analyses, ultimately increasing representation and equity in research, healthcare, and health.

To achieve this vision, CHWs developed a series of pragmatic steps to maximize their potential as full research team members (see Table 3). Steps include building relationships and articulating research roles, developing physical and programmatic infrastructure, establishing training programs, and initiating a CHW research career pathway.

**Table 3.** A plan to incorporate CHWs into the research team

Theme	Action Step
By bringing community context, insights, and relationships to all aspects of clinical research, CHWs can strengthen representation and equity in research, healthcare, and health	**1. Begin with fundamentals** Build relationships with CHWs that are genuine, respectful, and trusting Clearly articulate roles for CHWs and the research team Design studies so that CHWs are in an ongoing continuity relationship with study participants Compensate CHWs fairly **2. Develop Infrastructure** Designate a research hub, a physical location, (A) for research training, (B) for CHWs, community members, and researchers to build relationships, (C) for students to gain practical experience in research and in working with communities. Explore contracting with CHW services that are neighborhood-based. Ensure an accessible, community-focused mechanism for CHWs to obtain human subjects training **3. Provide Training** Provide study-specific training so that CHWs are well-prepared to recruit and assist participants Offer training in medical terminology, data, and epidemiology If relevant to the study, train CHWs in how to help participants navigate insurance issues **4. Build a Research Career Pathway** Develop a research certificate program for CHWs and interested community members Develop ongoing professional development in research for CHWs Establish scholarship programs for CHW professional development in research

## Discussion

This study examines clinical trial enrollment from the perspectives of Black community members and CHWs. We found that, in spite of decades of relevant research and policy, mistrust [15] continues to shape the perspectives of Black people towards clinical trial enrollment. Yet we also found that many community members and CHWs have a desire to better their community and the lives of future generations and see participation in trustworthy clinical trials as one way to do that.

These findings are consistent with research that finds, for multiple minoritized groups, altruism is an incentive for research participation [15], that Black people score highly on assessments of generative concern and generative acts [16], and that generativity, specifically the desire to improve one’s community, can be a response to the experience of racism [17]. The desire to build a better future may therefore be a meaningful point of entry into clinical research.

Previous research has identified CHWs as well-suited to recruiting racially minoritized research participants and to participation in research teams [18]. In our study, both CHWs and the community members with whom they work were enthusiastic about the potential for CHW engagement to make research relevant and accessible and to thereby increase participation. This research takes an important next step by outlining a pragmatic plan designed by and for CHWs to help build a CHW research pathway. Research into the skills and knowledge which CHW researchers require [19] provides a foundation on which this work can build. Including community members, such as CHWs, on the research team has the potential to both diversify clinical trial enrollment [8] and strengthen the community context of research [20].

This study has potential limitations. Participating CHWs had professional relationships with AVOC and may have been more favorably inclined towards study participation. In addition, our findings may be particularly applicable to CHWs who are trained and work in their community, where they may have a deeper understanding of local issues and a higher degree of community trust, compared to health system-trained CHWs. Finally, we did not collect data on age and cannot evaluate findings in a generational context. Further investigation, in different communities, is warranted to validate the findings and expand their implications for different settings.

Although mistrust in research, and healthcare at large, is ongoing among people who are Black, researchers may find common ground between their own interest in discovery and problem-solving and a potential sense of generativity among Black community members. Helping CHWs develop as researchers, particularly CHWs from the communities in which research is taking place, may be an important step to engaging Black community members, deepening research, and ensuring an inclusive research culture.

## Supporting information

10.1017/cts.2025.10221.sm001 Gotler et al. supplementary material Gotler et al. supplementary material

## Supplementary material

The supplementary material for this article can be found at https://doi.org/10.1017/cts.2025.10221.
