# Supplementary material for: A pragmatic plan to develop community health workers as researchers and strengthen Black clinical trial enrollment
Source: J Clin Transl Sci. 2025 Dec 17;10(1):e4. doi: 10.1017/cts.2025.10221 (PMC12797180; doi:10.1017/cts.2025.10221)
Supplement: Gotler et al. supplementary material [file S2059866125102215sup001.docx]

**Appendix A: Interview Questions**

Have you had any experience with medical research or heard about it from others?

What are your feelings about medical research?

One kind of research is called a clinical trial. Have you heard this term before?

Have you or someone you know had experience with clinical trial research?

If someone wanted to invite you to be part of a clinical trial, what information would you

want them to give you? What would help you decide to participate or not?

How would you want them to ask you? (In person? By mail or email? Another way?)

Do you think clinical trials can be important to people’s lives?

Are there problems with clinical trials? What are they?

Are there particular problems with clinical trials for people of color?

How would you feel about being a part of a clinical trial?

What would make you more likely to participate in a clinical trial?

What would make you less likely to participate?

Would payment of some kind change how you feel about participating in a clinical trial?

How much do you think people should be paid for participating in a clinical trial?

[CHW]: Do you think community health workers could be involved in recruiting and retaining clinical trial participants? How?

[CHW]: Do you think community health workers could be involved in planning or designing clinical trials? How?

[CHW] How would you feel about asking community members to be a part of a clinical trial?

[CHW] What type of training would you need to recruit people for a clinical trial?

[Community member] If you were thinking about signing up for a clinical trial, could a community health worker be helpful? What could they do to help?

What advice do you have for researchers who want to include people who are Black?

What else should we understand about participation in research by Black people?
